# Erratum: Identifying genuine protein–protein interactions within communities of gene co‐expression networks using a deconvolution method

**DOI:** 10.1049/syb2.12053

**Published:** 2022-10-29

The authors wish to bring to the readers' attention the following errors in the article by Jin Zhang and Shan Ju, ‘Identifying genuine protein–protein interactions within communities of gene co‐expression networks using a deconvolution method’.

In Section 5 Acknowledgements, one grant/award number was omitted. ‘XBS’ should be ‘XBS1822’.
